# Morgan County Medical Society

**Published:** 1868-11

**Authors:** C. J. Lucas

**Affiliations:** Secretary pro tem.


### MORGAN COUNTY MEDICAL SOCIETY.

The Society met in the Court House, Jacksonville, June 11th, at two o'clock P.M.

Upon being called to order by the President, Dr. David Prince, the proceedings of the last regular meeting were read and approved.

In the absence of the Secretary, Dr. C. J. Lucas was appointed Secretary *pro tem.*

The Treasurer of the last year, Dr. Craig, read his report, which was approved.

Dr. Robertson, of Tallula, exhibited a specimen of a tumor, taken from the cœcum and ascending colon, and gave the history of the case. The man, about 60 years of age, after a heavy lift in July last, felt an uneasiness in the bowels, which afterwards produced pain in that region. After a month, inflammation of the bowels set in, with formation of a tumor, which increased with the failure of his health. The tumor was hard and movable. Three weeks before his death he was troubled with diarrhœa in connection with a gurgling sound in the bowels after taking liquid, as is often heard in typhoid fever. One day before his death, May last, the tumor gave way, and convulsions followed. *Post mortem* examination showed an open tumor at the above-named place, with presence of pus in the bowels and opening of the tumor. The diagnosis was difficult during lifetime. The age and the slow progress of the disease could have given suspicion to cancerous disease, but no such diathesis in him or other members of the family could be traced.

Dr. Edgar, Sr., thought that formation of pus, if kept from the air, was not followed by early decline of health. Such tumors are common in women, in the region of the uterus and vagina, who are more subject to like injuries as in the above case, producing abscess in parts distant from the place of injury, and obstructing, by pressure of the tumor, the functions of the adjacent organs.

Dr. Prince was of the opinion that mere presence of pus had no influence upon the decline of health, but the mechanical pressure and malposition of the bowels. He then related an interesting snake story, in which he was consulted.

Dr. Edgar, Sr., exhibited a cancerous tumor of the osteoid variety which he removed from the scrotum of a gentleman of this county. It was interesting chiefly on account of its being a different variety from any ever brought before and discussed by the Society. He also spoke of a case of hæmatoid cancer located in the throat, which he had under treatment last year. He held that cancer structure was specific and peculiar, both in form and mode of life.

Dr. Prince remarked, "It is thought that it is a blood disease. Why is syphilis not of the same nature? In the latter disease the commencement is local as a pimple on some part of the body, unless transmitted from mother to offspring. The first manifestation of cancer is a local tumor, and before it becomes large and branches out, it produces no local cachexia. Cancer, if cut out early, will produce no bad effects. Removals of cancer of the lips are generally successful, and they seldom return. The infiltration of the poison comes afterwards." He did not consider cancer a blood disease at its first appearance. An operation in Dr. Robertson's case, he thought, would only have accelerated the death of the patient.

Dr. Reed was of opinion that the cause of cancer is an imperfect development of the lymphatic system, and not a blood disease. Cancer and obscure tumors can often be kept back and cured by a proper tonic and narcotic treatment. He spoke of a case of cancer in which removal by the knife was urgently recommended, but the patient objected to it. He improved under treatment of iron and quinine. In another case of scirrhus, the disease was removed by tonic treatment. He died from old age, and from an attack of fever.

Dr. Edgar, Sr., doubted this theory, and thought that if phthisis and syphilis were caused by derangement of the lymphatic system, and a child of syphilitic parents should not be nursed by the mother, the disease on the offspring would be caused by the poisoned blood, and not by the imperfect lymphatics.

Dr. Prince read a very interesting and able paper on convulsions, which caused a general discussion on this subject. He was requested by the Society to furnish a copy of it to some medical journal.

Dr. Bibb said that he was in the habit of using anæsthetics in convulsions, but lately, in four cases, tried the voltaic battery with good effect. He advises voltaic electricity in many cases of headache.

Dr. Lucas stated that in convulsions caused from irritation in the stomach, or from teething, mustard given for its emetic effect will be beneficial, producing vomiting only afterwards. If caused from irritation of the bowels, injections of warm water, to a great extent, even in infants one to two pints, will often relieve the disease as soon as the bowels are well washed out.

Questions arose in regard to the use of the voltaic battery in headache and other neuralgic diseases.

Dr. Craig said he used it with benefit in toothache.

Dr. Fisher relies more on nerve and blood tonics in neuralgia. He recommends, in protracted cases of cholera-infantum, an early nutritive and stimulating treatment, by which, he thinks, convulsions can often be kept back.

Dr. DeLeuw recommends, in convulsions of plethoric cases, the application of cold water to the head and spine; in anæmic cases, spirituous washings in connection with the internal use of valerianate of zinc, hyoscyamus, and musk. He reported also a case of poisoning by arsenic, treated by an emetic, albuminous drinks, and hydrated oxide of iron, resulting in recovery.

Drs. Reed and Robertson were appointed to read essays at the next meeting.

At five o'clock the Society adjourned.

C. J. LUCAS, M.D., Secretary *pro tem.*

